# Bacterial profile and antimicrobial resistance in diabetic foot ulcer infections: a 10-year retrospective cohort study

**DOI:** 10.1016/j.bjid.2025.104570

**Published:** 2025-07-31

**Authors:** Roberto Zambelli, Ana Flavia Santos, Larissa Resende Moreira, Hugo Miguel Ribeiro, Rodrigo Simões, João Murilo Magalhães, Priscila Constantino, Maria Clara Salomão, Cesar de Cesar Netto, Amanda Oliveira Leopoldino

**Affiliations:** a Faculdade de Ciências Médicas de Minas Gerais, Belo Horizonte, MG, Brazil; b Rede Mater Dei de Saúde, Serviço de Ortopedia, Belo Horizonte, MG, Brazil; c Rede Mater Dei de Saúde, Controle de Infecção Hospitalar, Belo Horizonte, MG, Brazil; d Duke University, Durham, United States

**Keywords:** Antibacterials, Foot ulcers, Amputations, Diabetic foot, Retrospective cohort

## Abstract

**Introduction:**

Diabetic Foot Infections (DFI) are severe complications of diabetes, often resulting in poor clinical outcomes, including amputations. The objective of this study is to identify the main pathogens causing infections in the diabetic foot ulcers, as well as the antibiotic resistance profile.

**Methods:**

This study included all patients treated for diabetic foot infections at a private tertiary hospital between 2013 and 2022. Demographic data, including age, sex, Body Mass Index (BMI), and the level of amputation were extracted from electronic medical records and collected for all patients. Microbiological and resistance patterns were evaluated following standardized protocols. Cases with incomplete demographic or microbiological data were excluded.

**Results:**

This retrospective cohort study analyzed data from 459 diabetic patients, among them, 337 patients with positive cultures were included, resulting in 507 culture results from surgical samples. Gram-negative bacteria accounted for 55.2 % of isolates, with *Enterobacterales* (41 %) and non-fermenters (14.2 %) being most prevalent. *Proteus* sp. (10.3 %) and *Escherichia coli* (8.3 %) were the most common Gram-negative organisms, with significant resistance to ESBL (15.4 %) and quinolones (29.3 %). Among Gram-positive cocci (43.6 %), *Staphylococcus aureus* (16.8 %) showed 21.1 % methicillin resistance, while *Enterococcus* sp. exhibited vancomycin resistance (7 %). Multidrug resistance was identified in 16 % of *Pseudomonas* sp. and 63.6 % of *Acinetobacter* sp., raising concerns about limited therapeutic options.

**Conclusion:**

The predominance of Gram-negative bacteria and high levels of antimicrobial resistance highlight the need for regular monitoring of local microbiological profiles. Targeted antimicrobial strategies can significantly reduce the morbidity associated with DFI and improve clinical outcomes in diabetic patients.

## Introduction

Diabetes is a major global health issue, affecting approximately 382 million people worldwide and projected to reach 592 million by 2025.^1^ This disease is a metabolic disorder in which glucose levels are abnormally elevated due to the malfunction of pancreatic β-cells in insulin action.^2^ One of the most severe and common long-term complications of poorly controlled diabetes is foot ulcers, affecting about 15 % of patients over their lifetime.^3^ These ulcers are the leading cause of hospitalization among diabetic patients and are associated with high morbidity, poor wound healing, increased mortality and risk of lower limb amputation, reduced quality of life and high costs.^2^^,^^4^

Even though Diabetic Foot Ulcers (DFU) are initially superficial lesions, they can progress to deep infections and osteomyelitis.^5^ Given that diabetes affects the immune, vascular, and neural systems, the progression of this condition can be faster and more severe, especially in the advanced age, peripheral artery disease and anemia.^6^

DFU account for approximately 80 % of non-traumatic lower limb amputations caused by diabetic complications worldwide.^7^ These amputations have a high mortality rate, with a 5-year survival rate of 41 % to 48 % for major amputations and only 59 % for minor amputations.^8^ In Brazil, between 2011 and 2016, the National Health System performed 102,056 amputation surgeries, with 70 % of these procedures involving individuals with diabetes.^9^

The indiscriminate use of antibiotics in the diabetic population is even more problematic, as the side effects of these medications can be more severe. Due to coexisting comorbidities such as renal failure, heart failure, microangiopathy, among others, the metabolism of these medications is altered.^9^^,^^10^ Therefore, dosing must be carefully managed to avoid reactions such as toxemia, gastrointestinal disturbances, and acute organ failure. Another relevant factor in the inadequate management of antibiotics is the promotion of biofilm formation in ulcers, which leads to the selection of increasingly resistant bacteria.^11^^,^^12^

Diabetic Foot Infection (DFI) can be either mono- or polymicrobial, with polymicrobial infections being more common with prior antibiotic use.^11^ This clinical condition has been thoroughly investigated, and a wide range of pathogens has already been isolated, with gram-positive cocci, especially staphylococci, being the most frequently isolated.^10^^,^^13^^,^^14^ However, other gram-negative organisms, such as *Enterobacter cloacae*, and *Proteus mirabilis* are also found, highlighting the need for a detailed analysis of the chronic nature and anatomical location of these infections.^5^^,^^10^^,^^13^

Bacterial resistance in DFI is a critical concern, as it complicates infection management, prolongs hospital stays, and increases morbidity, mortality, and healthcare costs.^1^^,^^12^^,^^14^, ^15^, ^16^ These infections, often caused by Multidrug-Resistant (MDR) organisms, limit effective therapeutic options, necessitating more expensive treatments^17^

The objective of this study is to identify the main pathogens causing infections in the feet of diabetic patients in a long-term cohort, as well as the antibiotic resistance profile of these bacteria. This data will assist in the appropriate selection of antibiotics and will be determining factors in the prognosis of such lesions, helping to avoid interventions with higher morbidity.

## Methods

This retrospective cohort study aimed to assess the microbiological and clinical profiles of diabetic patients with foot infections requiring surgical intervention. The study included diabetic patients who underwent surgical treatment for foot infections at a private tertiary hospital in Brazil between 2013 and 2022. All included patients had been diagnosed with foot infections secondary to ulcers. Ethical approval for the study was granted by the Institutional Ethics and Research Committee (report number 6.435.267).

For accurate sample selection, only diabetic patients who underwent surgical procedures coded under the Brazilian Hierarchical Classification of Medical Procedures code 3.07.29.34–3 – “Surgical treatment of plantar ulcers” – were included. Patients undergoing multiple surgical interventions were considered multiple times only if the subsequent procedure addressed a newly diagnosed ulcer, rather than a follow-up to a previously treated ulcer. Cases with incomplete demographic or microbiological data were excluded to minimize bias.

The study initially employed a convenience sampling approach, including all diabetic patients who underwent surgical procedures for foot infections at the institution over a 10-year period. To enhance the reliability of the findings, a sample size calculation was performed retrospectively. The calculation assumed an expected prevalence of 57 % for gram-negative pathogens in diabetic foot infections, based on data from previous studies.^15^^,^^16^^,^^18^ With a confidence level of 95 % and a margin of error of 5 %, the minimum required sample size for a study assuming an infinite population was determined to be 377 patients.

All patients included in the study were admitted through the emergency department, either directly from their homes or referred from lower-complexity healthcare facilities. Therefore, the infections were predominantly community-acquired at the time of hospital admission.

Demographic data, including age at the time of the intervention and Body Mass Index (BMI), were extracted from electronic medical records and collected for all patients. The level of amputation was also recorded, with procedures classified into minor amputations (those below the ankle, such as toe, ray, and transmetatarsal amputations) and major amputations (those above the ankle, including transtibial and transfemoral amputations).

Laboratory and microbiological culture data were retrieved from institutional reports, following standardized collection protocols. All cultures were obtained intraoperatively from deep tissue samples, following thorough surgical debridement in the operating room, in order to minimize contamination and ensure accurate identification of the infecting organisms. According to institutional guidelines, 3–5 samples were collected from each ulcer for microbiological analysis. Anaerobic cultures were not routinely performed during the study period and therefore were not included in the analysis. However, fungal isolates identified through standard aerobic culture methods were recorded and described when present. The isolates were tested to determine their bacterial resistance profiles, including resistance to beta-lactams, carbapenems, quinolones, and other commonly used antibiotics. The data were aggregated and analyzed to ensure comprehensive profiling. To evaluate the association between bacterial resistance profiles and clinical outcomes, patients were categorized into two groups: those infected with Multiresistant (MR) organisms and those with non-Multiresistant (non-MR) organisms. Five continuous clinical variables were compared between the groups: duration of hospitalization at first admission, number of surgical procedures, number of outpatient follow-up visits, time to definitive treatment, and level of amputation.

Descriptive analyses were performed to evaluate patient demographics, microbiological profiles, and patterns of antimicrobial resistance. Hypothesis testing for normality was applied to all outcome variables. Data analysis was conducted using Stata/IC 15.1 for Mac (College Station, TX, USA).

## Results

**Fig. 1:**
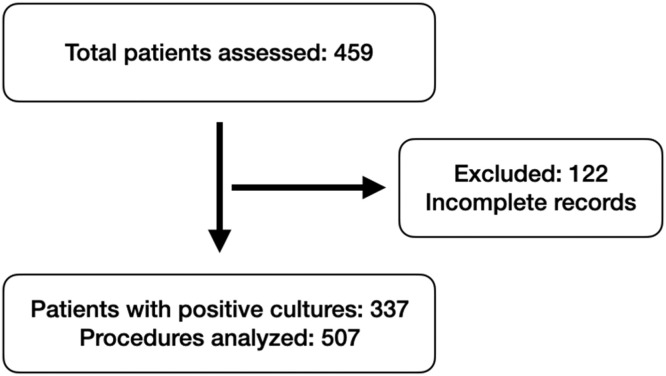
Flowchart of patient selection and microbiological analysis results.

A total of 459 diabetic patients were included in the study. Of the 337 patients with positive cultures who underwent surgery, a total of 507 culture results were included in the analysis (Fig. 1). The cohort was predominantly male (233 patients, 69.1 %), with a mean age of 66.4-years (range: 17–95 years, SD = 13.8) and a mean BMI of 27.1 kg/m^2^ (range: 15.6–42.5, SD = 4.7) (Table 1). Regarding surgical interventions, minor amputations were the most frequent procedures, accounting for 53.5 % of cases (180), followed by ulcer debridement (121 cases, 36.0 %). Major amputations, including transtibial and transfemoral levels, represented 10.5 % of the procedures (36 cases).

**Table 1:** Demographic characteristics of the studied population (*n* = 337).

**Characteristic**	**Mean / Count**	**Minimum**	**Maximum**	**Standard Deviation ( %)**
Age (years)	66.4	17	95	13.8
Weight (kg)	79.9	45	147	17.3
Height (m)	1.71	1.5	1.93	8.9
BMI (kg/m²)	27.1	15.6	42.5	4.7
Sex				
Male	233			69.1 %
Female	104			30.9 %
Level of amputation				
Ulcer debridement	121			36.0 %
Toe	50			14.9 %
Ray	92			27.2 %
Transmetatarsal	31			9.2 %
Chopart	7			2.2 %
Transtibial	30			8.8 %
Transfemoral	6			1.8 %
Total	337			100.0 %

**Fig. 2:**
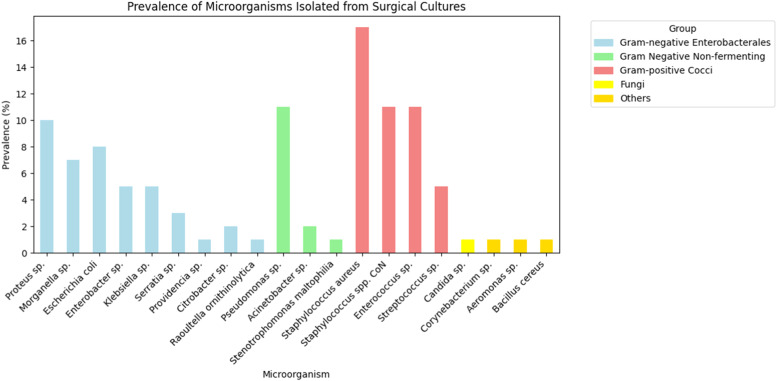
Prevalence of microorganisms isolated in surgical cultures.

Polymicrobial infections were observed in 135 of the 337 patients with positive cultures, corresponding to a rate of 40 %. Among the microorganisms isolated, 221 (43.6 %) were Gram-positive cocci and 280 (55.2 %) were *Enterobacterales* and non-fermenting gram-negative bacteria (Table 2). Additionally, 3 fungus and 3 other bacteria were found, representing 1.2 % of the total collected. The only fungus found was *Candida* sp. (0.6 % ‒ *n* = 3), and the other microorganisms were *Aeromonas* sp., *Bacillus cereus* and *Corynebacterium* sp. (0.6 % ‒ *n* = 3) (Fig. 2).

**Table 2:** Prevalence of microorganisms isolated in surgical cultures.

**Type**	**Microorganism**	**Prevalence ( %)**	**Number (n)**
Gram-negative (Enterobacterales and non-fermenting)	*Proteus* sp.	10 %	52
	*E. coli*	8 %	42
	*Morganella* sp.	7 %	35
	*Klebsiella* sp.	5 %	26
	*Enterobacter* sp.	5 %	25
	*Serratia* sp.	3 %	15
	*Citrobacter* sp.	2 %	9
	*Providencia* sp.	0,6 %	3
	*Raoultella ornithinolytica*	0,2 %	1
	*Pseudomonas* sp.	11 %	56
	*Acinetobacter* sp.	2 %	11
	*Stenotrophomonas maltophilia*	1 %	5
Gram-positive cocci	*Staphylococcus aureus*	17 %	85
	*Enterococcus* sp.	11 %	57
	Coagulase-Negative Staphylococci (CoNS)	11 %	56
	*Streptococcus* sp.	5 %	23
Fungus	*Candida* sp.	0.6 %	3
Other less frequent	*Aeromonas* sp., *Bacillus cereus, Corynebacterium* sp.	0.6 %	3

Among the Gram-negative bacteria, the group with the highest incidence was *Enterobacterales* at 74.2 % (208/280), with the following bacteria: *Proteus* sp. (52), *Escherichia coli* (42), *Morganella* sp. (35), *Klebsiella* sp. (26), *Enterobacter* sp. (25), *Serratia* sp. (15), *Citrobacter* sp. (9), *Providencia* sp. (3) and *Raoutella ornithinolytica* (1). Another 25.8 % (72/280) of Gram-negative bacteria were non-fermenting, including *Pseudomonas* sp. (56), *Acinetobacter* sp. (11), and *Stenotrophomonas maltophilia* (5) (Fig. 2).

**Fig. 3:**
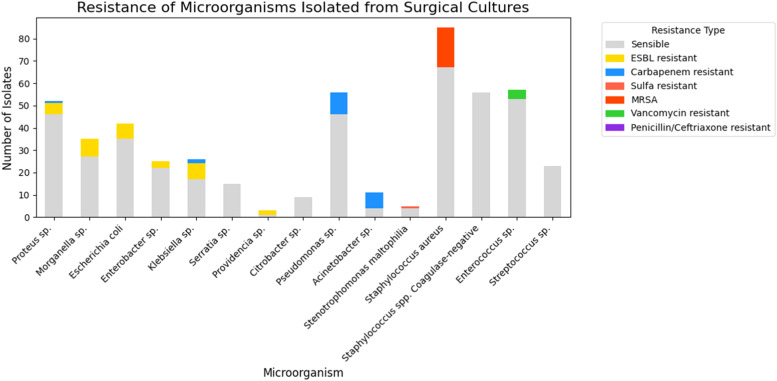
Resistance of microorganisms to antibiotics.

In an in-depth analysis of Enterobacterales, it was observed that only 1.4 % (3/208) were Carbapenem-resistant (*Proteus* sp.^1^ and *Klebsiella* sp.^2^) and 15.4 % (32/208) were ESBL-producing bacteria (Extended Spectrum Beta-Lactamase): *Morganella* sp. (8), *Klebsiella* sp. (7), *E. coli* (7), *Proteus* sp. (5), *Enterobacter* sp. (3), *Providencia* sp. (2). Among other classes of antibiotics, 29.3 % (61/208) of *Enterobacterales* were resistant to quinolones and 18.2 % (38/208) were resistant to sulfamethoxazole-trimethoprim (Fig. 3).

Regarding non-fermenting gram-negative bacteria 22.2 % (16/72) were Carbapenem-resistant, representing 63.6 % (7/11) of the *Acinetobacter sp*. and 16 % (9/56) of the *Pseudomonas* sp. Only one strain was Polymyxin resistant (1.3 % ‒ 01/72) and it was *Acinetobacter sp*. In relation to *Pseudomonas* sp. 26.7 % (15/56) were resistant to cephalosporin and/or piperacillin-tazobactam,19.6 % (11/56) to quinolones and only 3.5 % (2/56) to amikacin. Finally, for *Stenotrophomonas maltophilia*, of the 5-strains, 1 was resistant to sulfamethoxazole-trimethoprim (Fig. 3).

Among gram-positive cocci bacteria the most prevalent were *Staphylococcus aureus* (85), followed by *Enterococcus* sp. (57), Coagulase-Negative Staphylococci (CoNS) (56), and *Streptococcus* sp. (23) (Fig. 2). Regarding resistance, for *Staphylococcus aureus* 21.1 % (18/85) were Methicillin-Resistant *Staphylococcus Aureus* (MRSA), 45.8 % (39/85) resistant to clindamycin, 17.6 % (15/85) to quinolones and all strains were sensitive to sulfamethoxazole-trimethoprim. For Coagulase-Negative Staphylococci (CoNS) no resistant to vancomycin was observed, but resistance to quinolones 60.7 % (34/56) and to clindamycin 53.5 % (30/56). Vancomycin-Resistant *Enterococcus* (VRE) were identified in 4 of 57 strains (7 %), and among them only one was resistant to amoxicillin/ampicillin. And finally, among *Streptococcus* sp., no resistance to penicillin or ceftriaxone has been identified (Fig. 3).

**Fig. 4:**
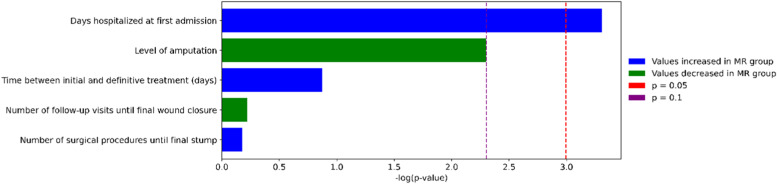
Association between multiresistant infections and clinical outcomes.

To explore the association between bacterial resistance and clinical outcomes, we compared five clinical variables between patients infected with Multiresistant (MR) and non-Multiresistant (non-MR) organisms. Among these, only the duration of the initial hospital stay showed a statistically significant difference (*p* = 0.037), with MR patients experiencing longer admissions. The level of amputation approached marginal significance (*p* ≈ 0.10), suggesting a possible trend toward more severe outcomes in the non-MR group. No significant differences were found for the number of surgical procedures, follow-up visits, or time to definitive treatment. These comparisons are illustrated in Fig. 4.

## Discussion

Diabetic foot infections are commonly polymicrobial and frequently involve antimicrobial-resistant organisms, which complicates treatment and increases the risk of poor outcomes. In our cohort, Gram-negative bacteria were slightly more prevalent than Gram-positive cocci, reflecting a pattern that has been increasingly reported in diabetic foot infections worldwide. *Staphylococcus aureus* (16.8 %), with 21.2 % of cases being MRSA, was the most prevalent bacterium found overall and among Gram-positive isolates, followed by *Enterococcus* sp. (11.2 %) and coagulase-negative *Staphylococcus* spp. (11.1 %), including one case of VRE. Among Gram-negative bacteria, *Proteus* sp. (10.3 %) and *E. coli* (8.3 %) were the most prevalent, with significant resistance to ESBL, ciprofloxacin, and sulfamethoxazole/trimethoprim. Non-fermenters, such as *Pseudomonas* sp. (11.1 %), demonstrated high rates of multidrug resistance (16.1 %), highlighting the importance of continuous local surveillance to guide empirical therapy in these complex infections.

DFUs, of multifactorial etiology, typically develop due to extrinsic mechanical factors, such as high plantar pressures and local trauma, combined with intrinsic factors, including peripheral neuropathy, peripheral arterial disease, and deficiencies in the immune response of the patient.^3^, ^4^, ^5^ When infection is not detected and treated early, it can spread from superficial tissues to deeper structures.^16^ In this cohort, we included only diabetic patients hospitalized for the treatment of foot infections, with a high incidence of minor (53.5 %) and major (10.5 %) amputations, highlighting the severity of diabetic foot infections.^6^

DFI can be mild, moderate, or severe, and may be mono- or polymicrobial. The pathogenic organisms involved in the infection vary depending on the location of the lesion, the duration of the disease, the patient's lifestyle, socioeconomic conditions, and prior antibiotic use.^11^ Our sample revealed a variety of over 15 microorganisms, with a predominance of Gram-negative bacteria, particularly *Enterobacterales*, in 55.2 % of the samples, aligning with findings reported in the literature.^12^^,^^15^^,^^16^ The diversity of these pathogens is an important contributor to the chronicity and severity of DFUs.^5^

Another critical factor to highlight is the profile of multi-resistant bacteria. Of all the samples analyzed, 14.3 % (73/507) were classified as multi-resistant. Among Gram-positive cocci, *Staphylococcus aureus* exhibited the highest prevalence of resistance (21.1 %), a finding consistent with several reference studies, which varies between 16.78 %‒18 %.^1^^,^^14^^,^^19^ In one Brazilian study, 17 samples (50.0 %) were positive for *S. aureus* among the 34 analyzed, and five isolates (29.4 %) were characterized as MRSA strains.^14^ Due to the absence of systematically recorded treatment response data, we were unable to assess whether infections caused by resistant organisms resulted in therapeutic failure or required changes to the initial antimicrobial regimen. This underscores the urgent need for continuous monitoring and the clinical relevance of implementing targeted antimicrobial protocols to address these challenges effectively.^1^^,^^5^^,^^10^^,^^16^^,^^20^

Among Gram-negative bacteria, the highest prevalence of resistance was observed in ESBL-producing *Enterobacterales* (15.4 %) and carbapenem-resistant non-fermenting Gram-negative bacteria (22.2 %). These results are lower than other studies, which presented ESBL prevalence as high as 60 %.^18^ Additionally, 29.3 % of isolates showed resistance to quinolones, while 18.2 % were resistant to sulfamethoxazole-trimethoprim. In cases of severe infections or when dealing with carbapenem-resistant organisms, combination therapy may be beneficial. This could involve using a polymyxin in combination with a β-lactam or aminoglycoside to enhance efficacy and reduce the risk of resistance development.^21^

While the reviewed literature reports MDR prevalence rates as high as 93 %.^5^^,^^10^^,^^11^^,^^13^^,^^15^^,^^16^ our findings demonstrated a comparatively lower overall prevalence of MDR bacteria. MDR was defined as non-susceptibility to at least one agent in three or more antimicrobial categories.^22^ However, at least 16 % of *Pseudomonas* sp. isolates and up to 63.6 % of *Acinetobacter* sp. isolates were classified as MDR. This finding is particularly concerning, as these bacteria are associated with severe infections, higher rates of therapeutic failure, and worse clinical outcomes.^16^^,^^21^^,^^23^ Continuous monitoring of antimicrobial susceptibility patterns is crucial for effective management and treatment of infections caused by these resistant organisms.^21^

A Brazilian study revealed that the most used empirical antibiotics for soft tissue infections (DFI) are amoxicillin-clavulanate, ampicillin-sulbactam, and the combination of ciprofloxacin with clindamycin.^24^ In the sample from this study, these empirical regimens faced significant limitations due to the high prevalence of resistance in Enterobacterales (15.4 % ESBL), *Pseudomonas* sp. (19.6 % resistant to quinolones), and *Staphylococcus aureus* (45.8 % resistant to clindamycin and 21.1 % MRSA). Therefore, institutional protocols must remain consistently updated with the specific microbiological profile of each institution to optimize empirical coverage.^17^^,^^21^ Although most infections in our sample were likely community-acquired, as patients typically arrived from home or were referred from lower-complexity facilities, it is possible that some had received prior treatment or hospitalization elsewhere, which may have influenced the resistance profiles observed. This scenario reflects the real-world trajectory of diabetic patients with complex infections and reinforces the importance of continuous microbiological surveillance.

This study presents significant strengths. It is a retrospective cohort spanning a 10-year period, including diabetic patients with positive cultures and detailed data on microbiological profiles and antimicrobial resistance patterns in diabetic foot infections treated at a tertiary center. Furthermore, all patients underwent surgical treatment performed by the same team, following strict and well-structured protocols, which enhanced the uniformity of the approach and case management, allowing for a more accurate assessment of the included sample. However, due to the retrospective nature of data collection and the exclusion of cases with incomplete records (although necessary to minimize bias) some information may not have been adequately documented. In particular, mortality data were not consistently available, preventing the analysis of associations between MDR infections and patient death.

Despite detailed treatment data were not recorded for analysis, it is standard practice in our institution to initiate empirical antibiotic therapy after sample collection and before culture results are available. This empirical regimen is defined based on the microbiological profiles of previous patients, which are reviewed periodically. Once culture results are finalized, treatment regimens are reassessed and adjusted to ensure targeted antimicrobial coverage.

Although the sample size calculation estimated the need for 377 patients to ensure statistically robust analyses, our final sample included 337 patients, slightly below the ideal number. This discrepancy is attributable to the retrospective nature of the study and the inclusion of data from a single center over a 10-year period. Nevertheless, the difference between the achieved and calculated sample sizes is relatively small, and the effective sample size was sufficient to identify significant trends in microbiological profiles and antimicrobial resistance.

## Conclusion

The data presented in this study reflect a well-defined cohort of patients with homogeneous characteristics and highlight the importance of regular analysis of the bacterial profile in patients with DFI to ensure the efficacy of empirical antibiotic therapy. A predominance of Gram-negative microorganisms was observed compared to Gram-positive, with a considerable prevalence of multidrug resistance among some isolated pathogens. These findings confirm the need for infection control strategies and a more judicious use of antibiotics, especially in diabetic patients, whose condition predisposes them to more severe and resistant infections. The creation of institutional protocols based on the microbiological profile can significantly improve the clinical management of DFI, promoting more targeted and effective therapies.

## Funding

This study was supported by the Postgraduate Program of the Faculty of Medical Sciences through a postdoctoral research grant and by the 10.13039/100009565International Bone Research Association (IBRA) under its Scholarship Program C. The funding sources had no role in the study design, data collection, analysis, or interpretation, or in the decision to submit the manuscript for publication.

## CRediT authorship contribution statement

**Roberto Zambelli:** Conceptualization, Formal analysis, Validation, Methodology, Writing – original draft, Writing – review & editing. **Ana Flavia Santos:** Formal analysis, Validation, Methodology. **Larissa Resende Moreira:** Data curation, Writing – original draft. **Hugo Miguel Ribeiro:** Data curation, Writing – original draft. **Rodrigo Simões:** Conceptualization, Writing – review & editing. **João Murilo Magalhães:** Conceptualization, Writing – review & editing. **Priscila Constantino:** Data curation, Writing – original draft. **Maria Clara Salomão:** Data curation, Writing – original draft. **Cesar de Cesar Netto:** Validation, Writing – review & editing. **Amanda Oliveira Leopoldino:** Formal analysis, Validation, Methodology, Writing – review & editing.

## Conflicts of interest

The authors declare that they have no conflicts of interest related to this study. No financial or personal relationships exist that could influence the content of this work.
